# New Bills of Mortality for London

**Published:** 1841-07

**Authors:** 


### PART FOURTH.

### Medical Intelligence.

---

### TABLE OF MORTALITY FOR LONDON,

For the Fourth Quarter of 1841:

Showing the number of Deaths from all causes registered in twelve weeks, from the 2d January, 1841, to the 27th March, 1841.

| Causes of Death. | Total Deaths. |
|---|---|
| **Class I.** | |
| Smallpox | 588 |
| Measles | 150 |
| Scarlatina | 155 |
| Hooping-cough | 601 |
| Croup | 84 |
| Thrush | 42 |
| Diarrhœa | 61 |
| Dysentery | 16 |
| Cholera | 1 |
| Influenza | 121 |
| Typhus | 313 |
| Erysipelas | 83 |
| Syphilis | 4 |
| Hydrophobia | 0 |
| Total Epidemic, &c. | 2219 |
| **Class II.** | |
| Cephalitis | 156 |
| Hydrocephalus | 403 |
| Apoplexy | 217 |
| Paralysis | 233 |
| Convulsions | 709 |
| Epilepsy | 49 |
| Insanity | 14 |
| Delirium tremens | 12 |
| Dis. of brain, &c. | 138 |
| Total Dis. of Brain, &c. | 1931 |
| **Class III.** | |
| Quinsy | 19 |
| Bronchitis | 289 |
| Pleurisy | 25 |
| Pneumonia | 1214 |
| Hydrothorax | 74 |
| Asthma | 706 |
| Consumption | 1659 |
| Dis. of lungs, &c. | 271 |
| Total Dis. of Lungs, &c. | 4257 |

| Causes of Death. | Total Deaths. |
|---|---|
| **Class IV.** | |
| Pericarditis | 15 |
| Aneurism | 15 |
| Dis. of the heart, &c. | 249 |
| Total Dis. of Heart, &c. | 279 |
| **Class V.** | |
| Teething | 216 |
| Gastritis, enteritis | 199 |
| Peritonitis | 16 |
| Tabes mesenterica | 56 |
| Ascites | 4 |
| Ulceration | 19 |
| Hernia | 32 |
| Colic or ileus | 42 |
| Dis. of stomach, &c. | 72 |
| Hepatitis | 20 |
| Jaundice | 26 |
| Dis. of the liver, &c. | 93 |
| Total Dis. of Stom. &c. | 795 |
| **Class VI.** | |
| Nephritis | 9 |
| Diabetes | 10 |
| Stone | 3 |
| Stricture | 3 |
| Dis. of the kidneys, &c. | 38 |
| Tot. Dis. of Kidneys, &c. | 63 |
| **Class VII.** | |
| Childbed | 127 |
| Ovarian dropsy | 5 |
| Diseases of uterus, &c. | 41 |
| Tot. Dis. of Uterus, &c. | 173 |
| **Class VIII.** | |
| Rheumatism | 37 |
| Diseases of joints, &c. | 44 |
| Total Dis. of Joints, &c. | 81 |

| Cause of Death. | Total Deaths. |
|---|---|
| **Class IX.** | |
| Ulcer | 7 |
| Fistula | 5 |
| Diseases of skin, &c. | 2 |
| Total Dis. of Skin, &c. | 14 |
| **Class X.** | |
| Inflammation | 66 |
| Hemorrhage | 46 |
| Dropsy | 480 |
| Abscess | 42 |
| Mortification | 88 |
| Scrofula | 23 |
| Carcinoma | 78 |
| Tumour | 19 |
| Gout | 23 |
| Atrophy | 81 |
| Debility | 290 |
| Malformations | 13 |
| Sudden deaths | 227 |
| T. Dis. of Uncert. Seat. | 1476 |
| **Class XI.** | |
| Old age or nat. decay | 1182 |
| **Class XII.** | |
| Intemperance | 4 |
| Privation | 17 |
| Violent deaths | 284 |
| Total by Violence, &c. | 305 |
| **Class XIII.** | |
| Causes not specified | 43 |
| T. Deaths from all causes<br>Males 6373, Females 6445 | 12818 |

